# Thermally Driven Selective Nanocomposite PS-PHB/MGC Nanofibrous Conductive Sensor for Air Pollutant Detection

**DOI:** 10.3389/fchem.2018.00432

**Published:** 2018-09-25

**Authors:** Joshua Avossa, Emiliano Zampetti, Fabrizio De Cesare, Andrea Bearzotti, Giuseppe Scarascia-Mugnozza, Giuseppe Vitiello, Eyal Zussman, Antonella Macagnano

**Affiliations:** ^1^Institute of Atmospheric Pollution Research-National Research Council (IIA-CNR), Monterotondo, Italy; ^2^Department of Innovation in Biological Systems, Food and Forestry, University of Tuscia, Viterbo, Italy; ^3^Department of Chemical, Materials and Production Engineering, University of Naples “Federico II”, Naples, Italy; ^4^CSGI, Consorzio Interuniversitario per lo Sviluppo dei Sistemi a Grande Interfase, Sesto Fiorentino, Italy; ^5^Faculty of Mechanical Engineering, Technion - Israel Institute of Technology, Haifa, Israel

**Keywords:** electrospinning technology, mesoporous graphene, hybrid and nanocomposite polymer nanofibers, gas/VOCs conductive sensor, sensor working temperature effects

## Abstract

The potentials to use the working temperature to tune both the sensitivity and the selectivity of a chemical sensor based on a nanostructured and nanocomposite polymer layer have been investigated and described. Thus, in a single step, a peculiar chemical layer was grown up onto IDE (Interdigitated Electrode) microtransducers by electrospinning deposition and using a single-needle strategy. The 3-component nanofibers, obtained from a mixture of polystyrene and polyhydroxibutyrate (insulating thermoplastics) and a known concentration of mesoporous graphitized carbon nanopowder, appeared highly rough on the surface and decorated with jagged islands but homogeneous in shape and diameter, with the nanofillers aggregated into clusters more or less densely packed through the fibers. The resulting sensor was conductive at room temperature and could work between 40 and 80°C without any apparent degradation. As the fibrous sensing layer was heated, the current increased and the sensitivity to some classes of VOCs such as an oxidizing gas drastically changed depending on the working temperature. More in detail, the sensor resulted highly sensitive and selective to acetic acid at 40°C but the sensitivity fell down, decreasing by 96%, when the sensor operated at 80°C. On the other hand, although an increase in temperature caused a general decrease in sensitivity to the tested VOCs (with a maximum of 14, 81, and 78% for amine, acetone and toluene, respectively) and water vapors (with a maximum of 55%), higher temperature affected only slightly the amine permeation, thus modifying the partial selectivity of the sensor to these chemicals. Conversely, when the operating temperature increased, the sensitivity to the detected gas, NO_2_, increased too, reporting a ~2 ppb limit of detection (LOD), thus confirming that the temperature was able to drive the selectivity of nanocomposite polymeric sensors.

## Introduction

The problem of classifying and further quantifying the chemical compounds in the air on a real-time basis is very crucial for a broad variety of activities in various fields, such as for industry (Wilson, 2012), medicine (Fitzgerald and Fenniri, 2017), food and agriculture (Srivastava et al., 2017; Basheer, 2018), indoor (Mad Saad et al., 2017) and outdoor environmental pollution (Wilson, 2012; Rai et al., 2017; Szulczynski and Gebicki, 2017), safety monitoring, and homeland security^1^ (Sekhar et al., 2010). More recently a common aim has been based on the attempt to develop portable and easy-to-use monitoring instruments for rapid and inexpensive analysis also of complex matrices, i.e., headspaces containing various volatile and gaseous compounds, based^1^ on sensors arrays or matrices with broad and partially overlapping sensitivity to various chemicals (olfactory machines and electronic nose systems). Each sensor has to transduce chemical information concerning multi-component gaseous mixtures into a series of measurable signals (Andrzej and Maciejewsk, 2012). As a part of these systems, sensors are exploited independently and simultaneously, and then treated as independent sensing elements. Furthermore each sensor contributes to create a multivariate response, within a sensor array, of the analyzed matrix. In order to be effective and successful, all the sensors should be characterized by as much chemical diversity as possible, on the assumption that they have not to be highly selective toward any given analyte, but highly and differently sensitive to every chemical class. On the other hand, the concept of an array of chemical sensors that can be used for any application is now outdated due to a series of difficulties, like the creation of universal databases from trained sensor arrays and the need to make the sensor array more selective to specific applications where gases or VOCs (Volatile Organic Compounds) to be revealed are present in trace amounts. For instance, in metal-oxide based sensors, that have been the most investigated and commercially used sensors, the working temperature is the main responsible of their sensitivity as well as their selectivity. Thus the same sensors, working at different working temperature values in order to detect various chemical classes with different sensitivity and selectivity, can be designed to create an array or a part of a suitable array for defined applications. For instance, SnO_2_-based sensors can work between 25 and 500°C but their best sensing temperatures depend on the kind of gas to be detected (Zakrzewski et al., 2006): an optimal sensing temperature of a SnO_2_ sensor to reveal CH_4_ has been reported to be about 400°C while that for sensing CO has been 90°C,confirming that temperature could be an effective tuner of the sensing features. Obviously in the case of MeO_x_ semiconductors temperature is the main responsible parameter for the amount of *O*^−^ distribution on the sensors surface, which is necessary for them to work properly. Vice versa, sensors based on conductive polymers are designed to work at room temperature, in order to both avoid an eventual thermal degradation (Zampetti et al., 2011) of the chemical layer and to favor the adsorption and diffusion mechanisms of the analytes through the surface. Generally, the sensitivity and selectivity are commonly ruled by polymer chemical structure and arrangement within the sensing layer (Sołoducho and Cabaj, 2016). In the present study, the potentials to use the working temperature to tune both the sensitivity and the selectivity of a chemical sensor based on a “soft-matter” chemical sensor have been investigated and described. Therefore a nanostructured and nanocomposite polymer sensor has been designed, fabricated, and then exposed to different chemical classes of VOCs and one gas within a range of working temperatures, compatible with the thermal stability of the polymers, to ascertain that the temperature also could be a modulator of selectivity and sensitivity such as for metal-oxide based sensors. Obviously, in the case of polymer based sensors, the temperature changes are expected to affect different chemo-physical parameters of the sensing process. The chemical layer was organized as a non-woven fabric of polymeric nanofibers obtained by electrospinning technology (ES). This technology allows designing and fabricating continuous nano-microfibers when a high electrical field acts on a droplet of a polymer solution with sufficient viscoelasticity. In literature, electrospinning (ES) has been suggested as one of the most promising candidates among the various nanotechnologies for designing and creating highly sensitive and smart sensing materials, due to its cost-effectiveness, high production rate, and peculiarity of the resulting nanostructures (Macagnano et al., 2015). Commonly, several approaches have been used to impart sensing capability to nanofibers (Ding et al., 2010), such as using a polymer sensing material to electrospun nanofibers (Macagnano et al., 2011, 2016), incorporating sensing molecules into nanofibers (Han et al., 2013; Su et al., 2014), or putting sensing material on nanofiber surface via coating/grafting technique (Huang et al., 2012), etc. Conductive polymer nanofibers can be obtained by several strategies as for example blending more polymers where at least one is conductive or including conductive nanofillers along fibers.

In this paper, we present the potentials to use the working temperature to tune both the sensitivity and the selectivity of a chemical sensor based on a nanofibrous and nanocomposite polymer layer. Indeed sensors based on conductive polymers have been commonly designed to work at room temperature, to both avoid an eventual thermal degradation of the chemical layer and to favor the adsorption and diffusion mechanisms of the analytes through the surface. On the other hand, temperature can be a useful strategy for provisionally modifying the arrangement of both polymer chains and the hosted nanofillers, as well as the related electrical and sensing features. Specifically, the attention has been focused on the challenging goal of obtaining conductive fibers electrospun by a mixture of two insulating polymers, polyhydroxibutyrate (PHB) (Hankermeyer and Tjeerdema, 1999; Acevedo et al., 2018) and polystyrene (PS), hosting a conductive nanopowder of mesoporous graphitized carbon (MGC). The polymers were mainly selected for several features like their versatility (generally used to make a wide variety of consumer products), eco-compatibility due to their biodegradability (PHB) and recyclability (PS) (Wünsch, 2000; Uyar and Besenbacher, 2008) and good environmental resistance to thermal excursion. Indeed, both the polymers are classified as thermoplastics, i.e., they can be heated to their melting point (${\text{T}}_{\text{b}}^{\text{PS}}$: 240°C, ${\text{T}}_{\text{b}}^{\text{PHB}}$: 175°C), cooled, and reheated again without significant degradation. Further, they were both soluble in CHCl_3_, meaning that a unique and easily electrospun mixture could be provided, and insoluble in H_2_O, meaning that the resulting fibers could be exposed to a wide range of relative humidity percentages without undergoing structural changes. Exploiting some of the properties of electrospinning technique, nanofibers have been designed in order to be rough and porous, in order to increase the exposed surface and favoring gas and VOCs permeation. Thus, a mixing of two different organic solvents and a proper surfactant agent were used in the ES deposition mixture. Furthermore, a lot of polymer interfaces inside fibers were expected due to the incompatibility of the two polymers. About MGC, it was selected as the conductive nanofiller: its structure made of a single layer of sp^2^ carbon atoms bonded in a hexagonal honeycomb crystalline structure displays outstanding physical properties as high carrier mobility [up to 350·10^3^ cm^2^/(Vs)], thermal stability (Bolotin et al., 2008), high mechanical strength (Young's module: 1 TPa and fracture strength: 130 GPa) and large availability of specific surface areas^2^. All these features were expected to provide both conductivity and a higher mechanical strength to the nanofibers. Graphitized carbon nanostructures have also been greatly investigated for their sensing features (Wang et al., 2016). Depending on the kind of application and transducer, graphene and their derivatives have been used oxidized or reduced, doped with metal-oxides - or metal-nanostructures, biomolecules or conductive polymers, etc. (Sahiner and Demirci, 2017). For these materials, gas adsorption is primarily due to physisorption on their surfaces (van der Waals forces) that can be tuned by chemical functional groups. In literature has been proven that some gases as NO_2_ (Novikov et al., 2016), NH_3_ (Karaduman et al., 2017), H_2_O (Khomenko et al., 2015), and CO (Panda et al., 2016) can be detected by graphene-based sensors, too, with a partial selectivity. The latter has been explained supposing that the adsorption reactions with various gas molecules occurred at the surface of graphene, where the adsorbed molecules acted as donors or acceptors. Such a charge transfer changed carrier concentration in graphene and then the conductivity of graphene-based sensors. Depending on graphene layer arrangement and doping, a graphene based sensor can show a p-type or n-type semiconductor behavior (Meng et al., 2013). The nanofillers, adopted for being included within the planned sensor, were provided also of a mesoporous membrane that conferred a larger available surface area (50–100 m^2^/g) to the nanopowder as well as the potential to get high selectivity through effects of molecular size exclusion (137 Å average pore diameter)^2^. The resulting MGC arrangement within the electrospun polymer nanofibers was expected to depend both on graphene/polymers mass ratio and its affinity to the hosting polymers, as well as on all the parameters of the electrospinning process. Electrical parameters are related to the quality of the MGC distribution within fibers, additionally to the MGC amount. Therefore, a rearrangement inside the polymer nanofibers due to temperature changes is expected to tune electrical parameters such as the sensing properties of the fibrous layer, thus suggesting an easy and novel strategy of tuning of selectivity and sensitivity of the polymer based sensors.

### Materials

Mesoporous Graphitized Carbon Nanopowder, MGC (<500 nm), Hexadecyltrimethylammonium Bromide, CTAB (~99%), Polystyrene, PS (Mw = 192,000 g/mol), Chloroform (≥99%), Acetone (≥99.5%), Toluene (≥99.8%), Polyvinylpyrrolidone, PVP (Mw = 1,300,000 g/mol), Acetic Acid, AcAc (≥99%), n-Butylamine (99.5%), Poly[(R)-3-hydroxybutyric acid, PHB (natural origin) were purchased from Sigma-Aldrich. Ethanol (≥99.8%) was obtained from Honeywell-Fluka. All chemical were used without further purification. Standardized pure air (5.0) and NO_2_ (5.00 ppm in N_2_), were purchased from Praxair-RIVOIRA, Italy, and stored in cylinders. Interdigitated Electrodes (IDEs), provided by Micrux Technologies (Spain), were fabricated on glass substrate (IDE sizes: 10 x 6 x 0.75 mm, Pt/Ti electrodes, 120 pairs, 10 μm wide × 5 mm long × 150 nm thick, with 10 μm gap) and rinsed with soap and a “base piranha” mixture at 60°C for ~15 min, (3:1, v:v, ammonia water and hydrogen peroxide water solution) and finally with Milli-Q water (~18 MΩ cm) before any use.

### Electrospinning deposition

The electrospun dispersion was prepared by mixing two different solutions: one containing the matrix of the fibers (PS and PHB) called Sol1 and the other one, called Sol2, containing the carbon nanopowder dispersion stabilized by a small amount of PVP. Sol1 solution (1:0.1:0.3 = PS:PHB:CTAB, mass ratio) was prepared, first, solubilizing 450 mg of PS pellets into 9 mL of chloroform under magnetic stirring. After completely dissolution, 60 mg of PHB were added into the solution and mixed at 45°C for 2 h. Then CTAB 150 mg and 1 mL of ethanol were poured into the system and mixed overnight at 45°C under magnetic stirring. Sol2 dispersion (1:0.4 = MGC:PVP, mass ratio) containing 50 mg of MGC, 20 mg of PVP, 1.8 mL of chloroform and 0.2 mL of ethanol were mixed and then sonicated for ~2 h. Sol1 and Sol2 (1.2 mL and 36 μL, respectively) were mixed under magnetic stirring for 1 h (1:0.1:0.3:0.003:0.001 = PS:PHB:CTAB: MGC:PVP, mass ratio). The resulting dispersion was loaded into a glass syringe (1 cm long stainless steel tip) and connected to a syringe pump. The fibers deposition was carried out in a home-made ventilated clean box equipped at ambient condition. The electrospinning apparatus consisted of a high power AC-DC converter, a high voltage oscillator (100 V) driving a high voltage (ranging from 1 to 50 kV), a syringe pump (Model KDS 200, KD Scientific) and a rotating conductive pipe with a 45 mm diameter grounded collector. The fibrous layers were fabricated by applying 2.9 kV of electrostatic DC voltage between the syringe tip and the collector, at a pump feeding rate of 900 μl/h. Once the potential was applied, the polymeric dispersion jet coated the IDEs placed on the grounded rotating collector (800 rpm), opposite to the syringe pump, at the distance of 8 cm. Deposition time was fixed at 2 min in order to obtain a thin coverage of the electrodes and at 20 min for EPR (Electron Paramagnetic Resonance) analysis and water contact angle measurement.

### Material characterization

UV-vis spectrophotometer (UV-2600 Shimadzu) was used to collect UV spectra of MGC. Samples were prepared from dilution of the electrospun dispersion (10 μL in 1 mL CHCL_3_) or Sol2 (1 μL in 1 mL of chloroform).

EPR measurements were performed by using a X-band Bruker Elexys E-500 spectrometer (Bruker, Rheinstetten, Germany). Samples (in turn, ~1 mg of MGC and electrospun fibers mats peel off an aluminum foil subject to rotation and deposition for 20 min) were co-axially introduced in a quartz sample tube with a diameter of 4 mm and were measured at 25°C. EPR spectra were recorded at the following settings: 100 G as sweep width, 1,024 points as resolution, 100 kHz as modulation frequency and 1.0G as modulation amplitude. Preventively, the amplitude of the field modulation was checked to be sufficiently low to avoid detectable signal overmodulation. To record the final EPR spectra, the attenuation value was fixed to 15 dB and 128 scans were accumulated with the aim to improve the signal-to-noise ratio. The specific g-factor value was determined by inserting Mg/MnO powder, as internal standard (Yordanov et al., 1999) in the quartz tube with the analyzed samples.

Optical micrographs were captured by Leitz-Wetzlar (Metallux 708082) microscope, for the evaluation of the quality coverage of the fibers deposited onto the IDE.

Fibers morphological analyses were carried out by means of micrographs from Scanning Electron Microscopy (SEM) and Transmission Electron Microscopy (TEM). The electrospun nanofibrous fabrics deposited on thin SiO_2_ wafers and sputter-coated with gold in a Balzers MED 010 unit were analyzed for SEM by a JEOL JSM 6010LA electron microscope.

For Transmission Electron Microscopy (TEM), samples were attached onto carbon-copper grids and observed with a JEOL 1200 EX II electron microscope. Micrographs were captured by the Olympus SIS VELETA CCD camera equipped the iTEM software.

### Measurement setup

Chemoresistors were housed in a measurement glass chamber (~100 mL volume) and connected to an electrometer (Keithley 6517) capable of measuring their electrical parameters and sending data to a PC (LabVIEW Software). The current was recorded by applying potential values from −7.0 to 7.0 V in ten steps of 0.7 V at different temperatures (20, 40, 60, 80, 100°C). Currents values vs. applied voltage were used to calculate the resistance of the fibrous coated IDE and its correlation to the temperature. All the batches of the chemoresistors fabricated in different dates but keeping the identical deposition parameters reported the same electrical features confirming the reproducibility of the deposition technique (data not shown).

Dynamic sensor measurements were carried out at different temperature (40, 60, 80°C) using: (i) 4-channel MKS 247 managing up to four MKS mass flow controllers (MFC), set in the range 0–200 sccm (standard cubic centimeter per minutes); (ii) Environics S4000 (Environics, Inc.) flow controller, containing three MFCs supplying different flow rates (up to 500, 250, and 2.5 sccm, respectively), managed by its own software. Pure air was mixed and used as gas carrier. Different fluxes of NO_2_ withdrawn from a cylinder at fixed concentration were mixed to pure air in order to test the dependence of IDE responses on different NO_2_ concentrations. For evaluating the effect of water and VOCs vapors (acetone, toluene, acetic acid, and n-butylamine) on the sensor conductivity, different compounds concentrations in air were generated by a bubbler filled with liquid water or VOCs and mixed with the gas carrier (air).

## Discussion and results

**Figure 1:**
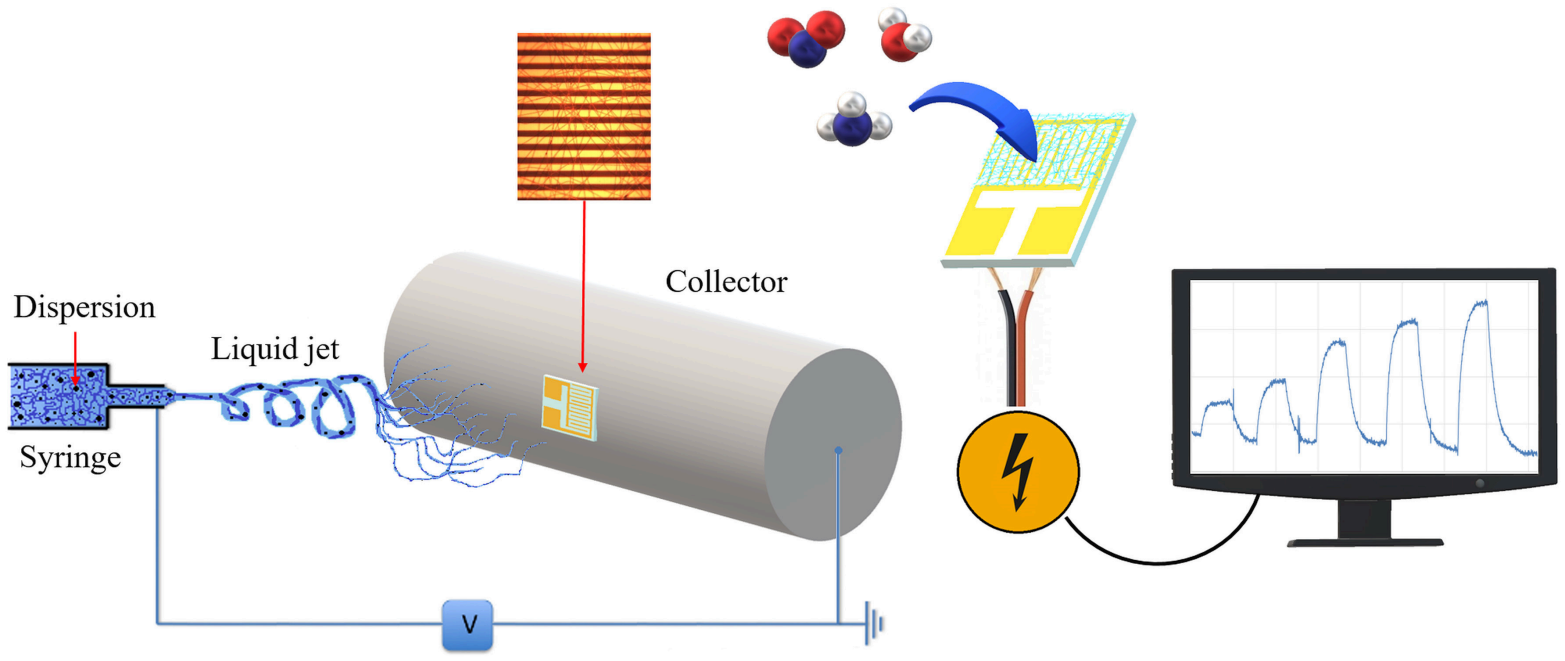
Sensor developing and usage scheme. The polymer mixture loaded in the syringe **(Left)** was electrospun by applying a voltage between the tip and the cylindrical collector where an interdigitated electrode is fixed and coated with the fibers. The coated IDE was used as transducer **(Right)** and connected to a PC for sensing current changes upon interaction with gases molecules.

**Figure 2:**
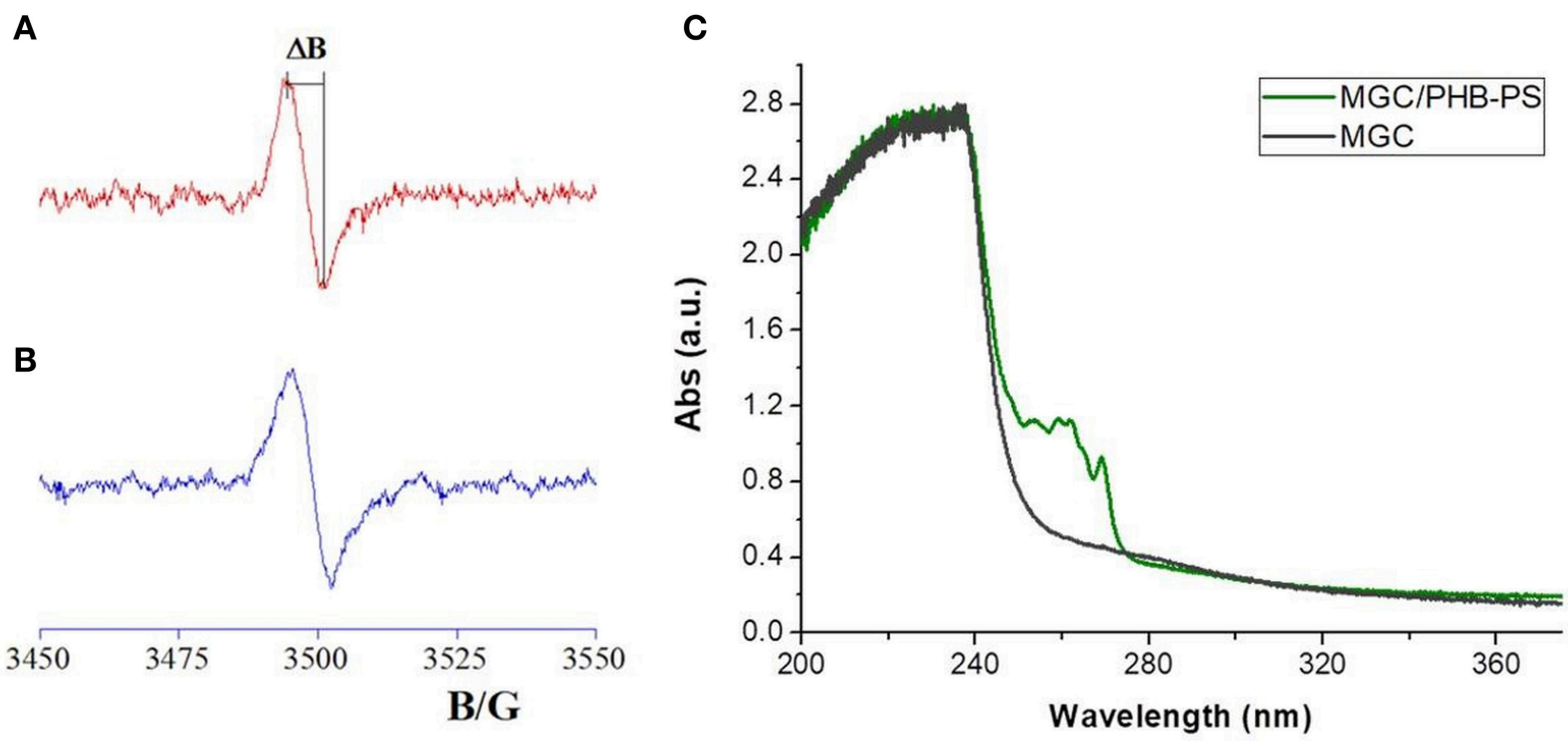
EPR spectra of MGC powder **(A)** and electrospun mats **(B)**. ΔB corresponded to the distance between the maximum and the minimum within the spectra. On the right **(C)** the UV-vis adsorption spectra of diluted polymer mixture of MGC/PHB-PS (light green) and diluted Sol2 in chloroform (dark green) are reported.

**Figure 3:**
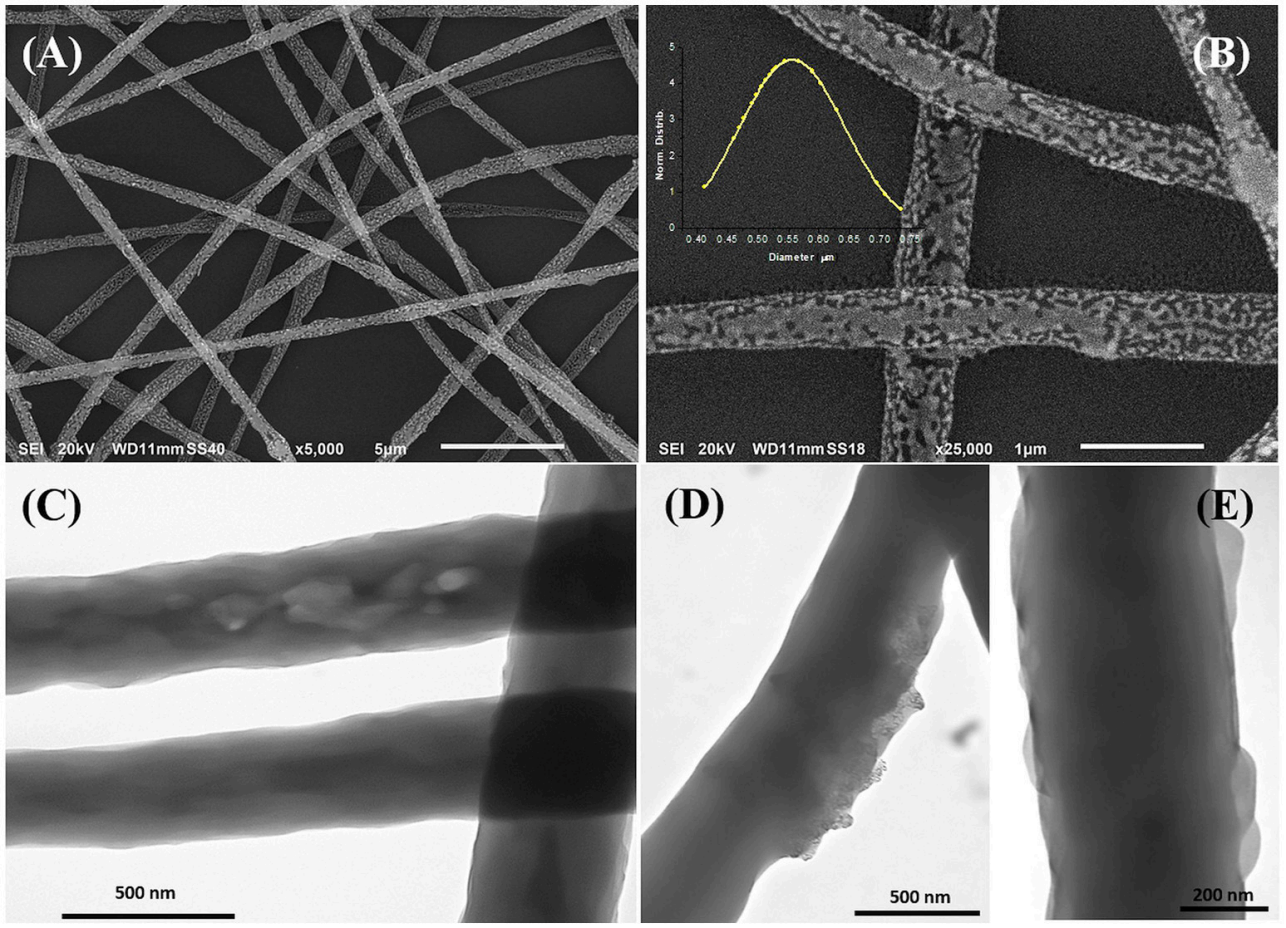
SEM **(A,B)** and TEM **(C–E)** micrographs of electrospun fibers at different magnifications. The inset in **Figure B** represents the diameter distribution of the fibers collected from the SEM micrographs. SEM micrographs showed cavities and roughness on the surface while TEM highlighted protrusions **(E)**, zones with different density **(C)**, and the MGC particles finely dispersed within the matrix **(D)**.

**Figure 4:**
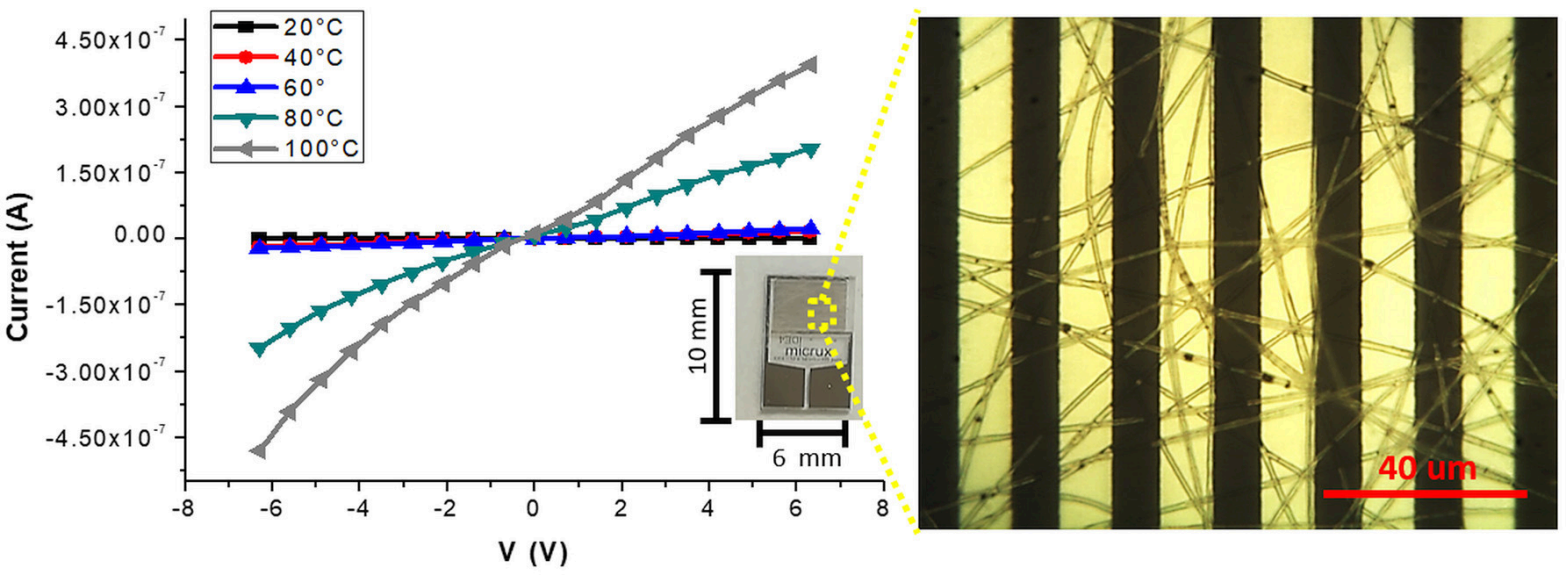
Current-Voltage curves at different temperatures (20, 40, 60, 80, 100°C) for the fibers coated IDE (inset) are plotted. On the right, an optical micrograph shows a homogeneous coverage of the transparent fibers onto the interdigitated platinum bars. The optical image highlighted the presence of sub-micrometric MGC clusters.

The nanocomposite fibrous layer was grown up by electrospinning deposition using a single-needle. A diagram depicting the sensor development and its working is shown in Figure 1. The polymer drop on the needle tip, comprising PHB, PS, and MGC was subjected to an electrical field generating electric charges on the liquid surface, then repulsive electrical forces, elongation between the needle tip and collector up to the solvent evaporation and fibers formation. The transducers can be fixed onto the grounded rotating cylinder in order to collect the ejected fibers on their electrodes and then be able to measure their electric and sensing features when exposed to the selected analytes. Despite the multiplicity of the components in the mixture, the electrospun traveling liquid jet stream proceeded without interruptions, micro-drops or nozzle fillings. All the substrates, i.e., IDEs, SiO_2_ wafers, and Al-foils, were fixed on the cylindrical collector and aligned within the deposition cone. After deposition IDEs and SiO_2_ wafers appeared coated with a thin fibrous fabric (the inset in **Figures 3A,4**, respectively). The heterogeneous system was collected for a few minutes on the interdigitated electrodes in order to link the metal fingers by a thin and highly porous film. Such fabrics resulted white/light gray, soft, and easy to peel off. Instead, a free-standing mat was got after at least 20 min of deposition onto the Al foil. After thermal incubation at 60°C for 12 h, the samples were investigated to outline their morphological, electrical, and sensing properties. The EPR spectra reported in Figure 2 depicted a single and very broad Lorentzian signal at a g-factor value of 2.0035 ± 0.0003, which is typical of carbon-centered radicals (Barklie, 2003; Kausteklis et al., 2011) and associable to paramagnetic defects within the graphitic structure (Cirić et al., 2009; Kausteklis et al., 2011; Tampieri et al., 2014). Any additional resonance that would belong to frequently observed transition-metal impurities was not revealed using magnetic field scanning over broader field-range (Kausteklis et al., 2011). A quantitative analysis of the EPR spectrum was also realized by determining the signal amplitude, ΔB. This parameter is directly obtained by the recorded spectra and is correlated to the mean distance between the radical centers (Vitiello et al., 2017), furnishing indirect information about the spatial distribution of paramagnetic centers within the whole material. For bare carbon nanopowder (Figure 2A), the EPR spectrum presented a ΔB = 6.3 ± 0.2 G. A similar signal was observed in the EPR spectra of the nanofibrous sample (Figure 2B), confirming the presence of MGC in the electrospun fibers (MGC-NFs). Here the ΔB value reported a slight increase with respect to the value obtained from the spectrum of MGC bare (ΔB = 6.7 ± 0.2 G), signifying that the radical centers are nearest and suggesting a more confined spatial distribution of MGC within the final electrospun materials. This result may be due to an aggregation of MGC inside the nanofibrous layer in domains with a thicker spatial density than in MGC powder. UV-Vis spectra (Figure 2C) of MGC (Sol2) and MGC in PHB-PS have the typical shape of graphene dispersion in chloroform except for the main peaks due to polystyrene absorbance (260 nm). The absorption band centered at 232 nm is ascribed to π-π* transitions of aromatic C–C bonds (signals in saturation). Instead the absorbance band at 269 nm is presumed to be due to the redshift of the graphene band due to the flakes dispersion and orientation within the polystyrene suspension (Khan et al., 2002; Çiplak et al., 2015; Uran et al., 2017).

The morphology of the electrospun fibers is summarized in Figure 3. The three-component nanofibers, obtained from the mixture of PS/PHB, that are immiscible polymers, and MGC, all diluted in a CHCl_3_ and EtOH, appeared extremely rough on the surface and decorated with jagged islands, which may indicate phase separation, but homogeneous in shape and diameter (d: 550 ± 170 nm). The long and continuous fibers as the beads absence suggested that an appropriated combination of electrospinning set of parameters was achieved (e.g., potential applied, nozzle-ground distance, the feed rate, chemical combination of solvents, viscosity, molecular weight, and structure of the carrier polymers, etc.). Therefore the resulting IDE coating was a highly porous network of nanofibers with interconnected void volumes (high porosity) and high surface-to-volume ratios (specific surface area) (Zampetti et al., 2013). Several parameters are expected to be the main responsible of the resulting fibers structure:

The use of an organic solvent combination (CHCl_3_: EtOH) whereas each component has a different rate of evaporation (T_b_: 61 < 78°C, respectively), viscosity (η: 0.57 < 1.20 cP, respectively) and relative polarity (0.259 < 0.654, respectively)^2^

The use of a mixture of chloroform and ethanol (9:1 v/v) that are solvent and non-solvent for the polymers respectively may induce a phase separation during the electrospun fibers formation. Qi *et al*. produced highly porous Poly(L-lactic acid) (PLLA) fibers by a solvent (dichloromethane) and nonsolvent mixture (Butanol), suggesting that the higher amount of nonsolvent and volatility difference between the nonsolvent and solvent the higher the porosity (Qi et al., 2009). In other works, the porosity was regulated by the humidity percentage: water (acted as a non-solvent for the polymer) diffused into the polymer jet solution causing phase separation and formation of porous fibers (Megelski et al., 2002; Pai et al., 2009).

The use of two polymers soluble in the same solvent (CHCl_3_) but incompatible with each other, hence separating in different domains. Zhong et al. formed a blend of polymers by electrospinning a solution of poly(ethylene oxide) (PEO) and PS, where PEO separated in smaller domains within the PS fibers (Zhong et al., 2011). Bognitzki et al. produced fibers possessing co-continuous phase morphologies resulted from phase separation processes occurring during fiber formation of Poly(L-lactic acid) and PVP (Bognitzki et al., 2001).

The introduction of a quaternary ammonium surfactant capable of increasing polymer surface roughness. In fact, CTAB salt, increasing the charge density of the polymeric solution, can affect the average fiber diameter such as the presence of crystalline particles, can increase the surface roughness (Sarac, 2016).

**Figure 5:**
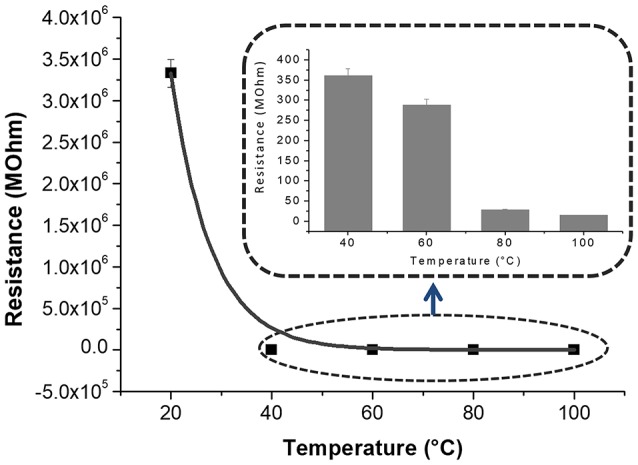
Resistance-Temperature data and corresponding interpolating curve are reported. The inset showed the resistance values at 40, 60, 80, and 100°C in a smaller scale.

Figures 3C–E, is a composition of TEM micrographs of fibers details, confirming an irregular edge of the fibers, with plicas and globosity. Since the mass ratio between PHB and PS is 0.13 and due to their immiscibility, the resulting fibers might be composed with a matrix of PS hosting a randomly dispersion of PHB in the form of small aggregates (Figure 3C), that may be enhanced by the clearest areas along the fibers (lighter gray). Due to the rapid stretching of the electrified jet and the fast solvent evaporation, polymer macromolecules are forced to be oriented in the direction of elongation. This rapid event is able to inhibit the polymer chains from going back to their equilibrium conformations. As a result, electrospun nanofibers are featured by a high degree of molecular orientation. Thus PHB and PS chains were subjected simultaneously to the same electric field strengths, but since they are basically incompatible, the stretching of the PHB chains is probably limited by the incompatibility with the PS ones, as reflected by their phase. Indeed, the phase separation was distinctly apparent with small aggregates of a polymer confined in the immiscible matrix. About the distribution of MGC along fibers, due to the low contrast between MGC sheets and the polymer matrix, it is difficult to observe the MGC networks. At higher TEM magnification darker areas appear to be finely distributed within the fiber (darker areas, Figure 3D) without assembling into beads, but aggregated into clusters more or less densely packed through the fibers, where some of them protruded from the fibers. According to the MGC description provided by the supplier^2^, they had a sheet configuration with an approximate diameter of 35 nm, forming aggregates in the 175 nm size range and then agglomerates in the 400 nm range. Further, MGC had a 137 Å average pore diameter. The resulting fibrous layer, comprising a lot of interfaces as well as the rough fibers and pores, was expected to be an intriguing system for the development of chemical sensors, due to both the wide adsorption surface and the surface energy potentials involved. The microtransducers coated with the 2 min-deposited fibers and placed on a customized micro-heater of alumina were able to measure the electrical parameters of the resulting chemoresistor at increasing temperature values up to 100°C. Optical microscope pictures confirmed the formation of optically transparent polymeric fibers through which the electrodes of the underlying substrate can be visualized. A few of significant black MGC aggregates was found inside fibers suggesting that the graphene distribution was not completely homogeneous and probably affected too by the immiscibility of the two polymers (Figure 4, inset). Current-Voltage curves displayed a quasilinear relationship between the current changes and the increasing imposed voltage values. Such a chemoresistor reported a resistance value (R) of about 3.33·10^6^ MΩ when it worked at 20°C. Obviously, electrical conductivity was strictly related to the MGC content. Changing the amount of MGC the resistance value dramatically changed (data not shown). The MGC concentration here described (0.93% mass percentage) is related to the fibrous layer containing the minimum concentration that allowed the generation of stable and reproducible electrical signals at room temperature (close to the percolation threshold). At room temperature, whereas the carbon nanopowder is dispersed inside fibers forming a non-contact mode networks, the tunneling of electrons is expected to dominate the conduction of the composite system according to the percolation theory. Such a theory (Kirkpatrick, 1973; Mutlay and Tudoran, 2014) proposes that below a critical concentration, conducting fillers are individually isolated in an insulating polymer. As the concentration of the nanofillers increases, an inter-connected network of particles distributed inside the matrix is formed. This arrangement makes the material to sharply change from insulator to semi-metal conductor. Beyond the critical concentration (percolation threshold) all the particles appear divided only by a thin polymer layer that allows the quantum mechanical tunneling. Further concentrating the particle network will generate saturation and conductivity achieves an upper limit which is quite below the conductivity of pure nanofillers (metal-like). Alekseev et al. reported that, when conductive fillers are incorporated into polymer matrices, both their state of dispersion and their orientation are also significant for determining electrical conduction of composites (Alekseev et al., 2012). The relative high resistance value of MGC in PS-PHB nanofibers suggest that the graphitic powder was organized into a network allowing the fibrous layer being conductive. Further, I-V curve shape seems to be affected by the metal-like conductivity occurring within the “darker dots” where MGC were more densely packed. However, when the transducer was microheated up to 100°C, the chemoresistor reported a nonlinear increase in the current values (resistance decrease). Specifically, R abruptly decreased 9·10^3^ times, i.e., from 3.33·10^6^ to 3.61·10^2^ MΩ, when temperature was increased from 20 to 40°C. The chemoresistor reached 14 MΩ resistance when the working temperature was set at 100°C (Figure 5). The temperature dependence of conductivity was non-linear but showed always a positive behavior upon heating. This result suggests the domination of the tunneling resistance in comparison with the contact one (Syurik et al., 2013) (Gao et al., 2018). The latter usually dominates in highly filled composites when physical contact between particles occurs, while the former is depending on the small dielectric barriers (insulating polymer) between the particles (Sheng, 1980). Polymer heating effects can be found in the chain reorientation due to a group rotation on the polymer backbone (Alexander, 1999), including phenyl group rotation in polystyrene (Tonelli, 1973). Such reorganization is supposed to affect the temperature dependence of conductivity due to strong π-π interaction existing between aromatic organic molecules and the basal plane of the graphene. According to Cao et al., graphene sheets inside polystyrene films are able to build thermodynamically unstable networks at higher temperatures, (Cao et al., 2009), thus improving the connectivity of the conductive networks and then enhance the electrical conductivity.

**Figure 6:**
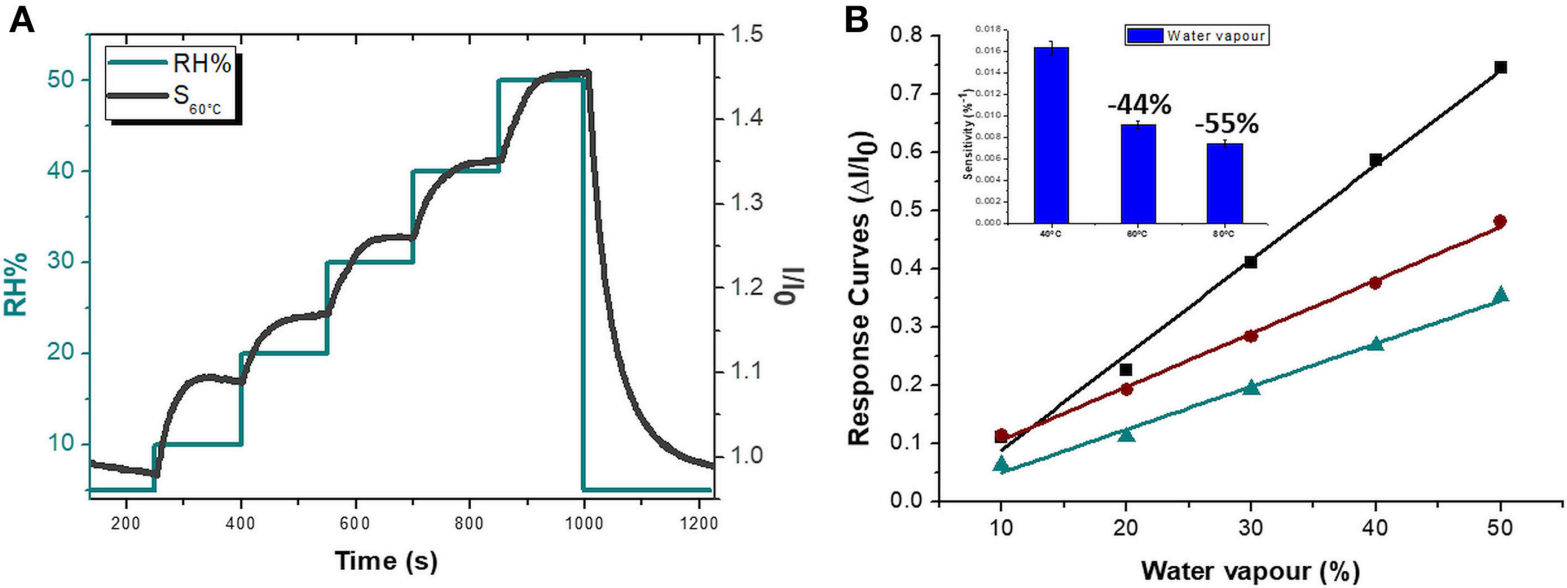
Sensor electrical features at 60°C (S60°C) in dependence on %RH: the Normalized current curves (I/I0, black) and Relative Humidity percentage (green) VS time are depicted **(A)**. Sensor Response Curves VS Relative Humidity percentage at 40, 60, 80°C (green, brown and black, respectively) with an inset showing the Sensitivity values at 40, 60 and 80°C are reported.

**Figure 7:**
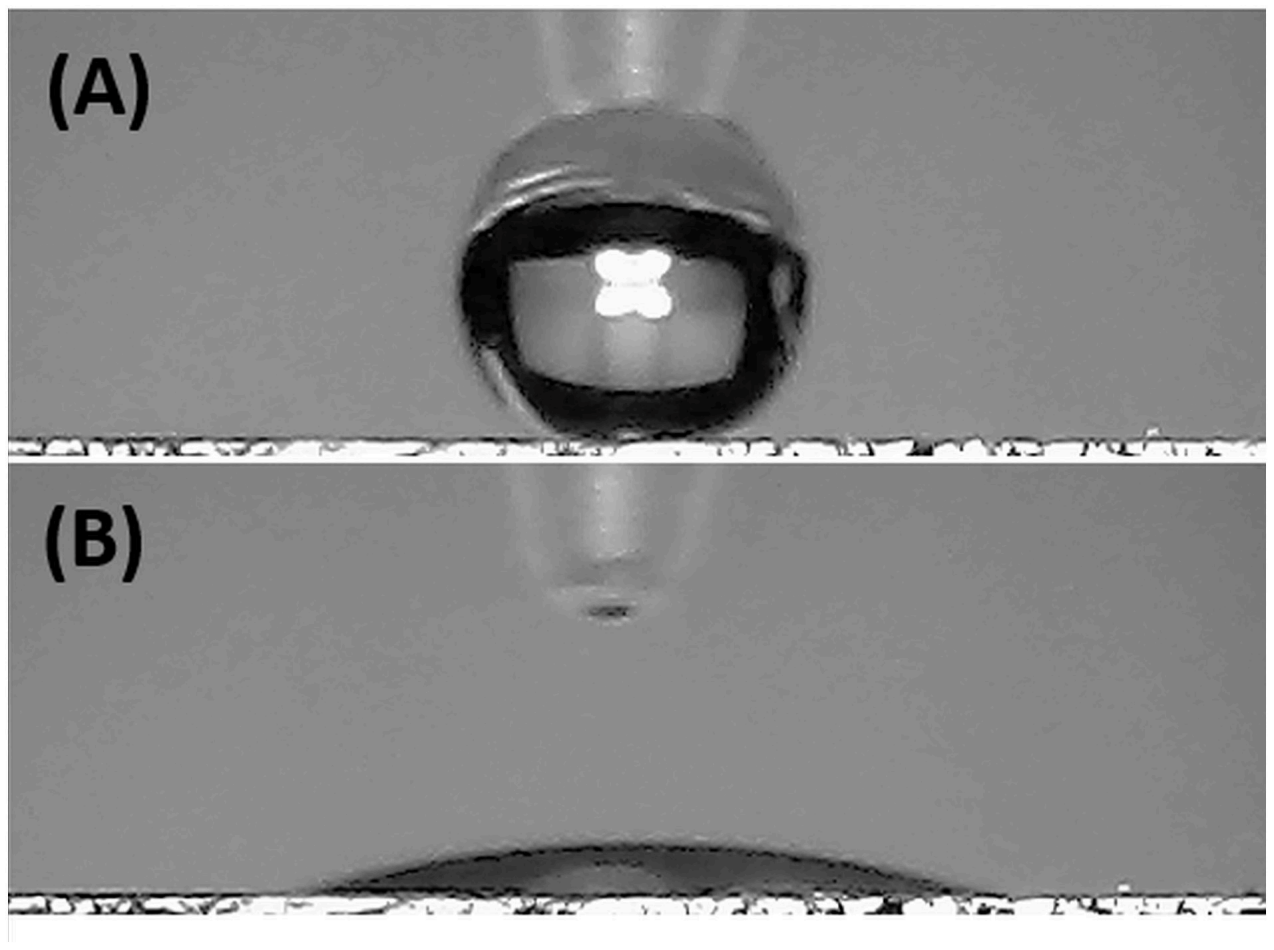
Pictures of a water droplet of 5 μL before **(A)** and after **(B)** touching the electrospun mat.

Since the electrical signal at room temperature was too noisy, all sensing measurements were carried out between 40 and 80°C. Measurements at higher temperature were avoided for limiting an eventual wear of the fibers. Standard pure air with increasing percentages of water vapors was flowed throughout the measuring chamber and the changes of the sensor current were reported in Figure 6A. The humidity is everywhere in the environment, thus it is crucial to control this parameter as well as to know its effects on the designed sensor. Indeed, water molecules in the air are commonly known as potential interfering agents in the interactions between the chemical sensors and the VOCs/gases. Measurements were ranging between dry and 50% humid air. A linear relationship between humidity and current changes was reported (Figure 6B), and, specifically, the current linearly increased when humidity percentage increased, too. When the T_w_ was set at 40°C and relative humidity was 50%, the normalized current increased by 7.46·10^−1^ times. Similarly, the same measurements were carried out with the sensor set at increased temperature values. As previously reported, the current increased linearly with the humidity, but the sensor sensitivity to water vapor decreased at higher temperature. Therefore the normalized response to 50% of relative humidity, calculated as *(I-I*_0_*)/I*_0_, where *I* is the current value due to the reaction with the analyte and *I*_0_ is the current value when sensor is under clean air, decreased to 3.58·10^−1^ from 7.46·10^−1^ when the T_w_ was set at 80°C. Measuring the sensitivity values at 60 and 80°C, they diminished of about 44 and 55%, respectively. Sensitivity, defined as the ratio of the incremental change in the sensor's output (Δy) to the incremental change of the measured in input (Δx), was calculated as the slope of the response curves (i.e., calibration curves) (Kalantar-zadeh, 2013). As a matter of fact, the fibers were designed to get a poor interaction with water molecules. Both the polymers were hydrophobic and planar structure of MGC should prefer π-π interactions, despite H-bonds due to structural defects and terminal carboxyl groups. Instead, the experimental results suggested that the sensor seemed sensitive to the relative humidity. Indeed, contact angle measurement (Figure 7) showed that once a droplet of water (5 μL) touched the surface of the ES mat (20 min deposition) a low water contact angle (~15°) was formed, demonstrating that fibers possessed an hydrophilic behavior, hence high affinity with water. More parameters were supposed to contribute to the unexpected sensor responses to the water. One of those is related to the porous and extremely wrinkled structure of the film (Zhang et al., 2012). Another one is related to the presence of numerous interfaces between MGC and the polymers (Pierleoni et al., 2018) as well as the two immiscible polymers, which facilitate the molecules permeation. Furthermore, since MGC has been designed with a mesoporous structure, it should easily entrap water molecules and interact by the oxygen atoms making part of the framework of each MGC sheet. Indeed, Boehm in his review stated that carbon black and other forms of carbon possessed a partial oxidized surface (Boehm, 1994). Finally, the salt used to allow the mixing of such a heterogeneous ES suspension, being a water soluble cationic surfactant, should favor the adsorption of H_2_O molecules on to the fiber surface. On the other hand, the general decreasing in sensitivity to water molecules due to the increasing in temperature should be related to the reduction of adsorption on the nanostructured material surface and the lowering the molecules diffusion due to the backbone motion of the polymer chains (Haslam et al., 1924; Ramesh and Duda, 2000).

**Figure 8:**
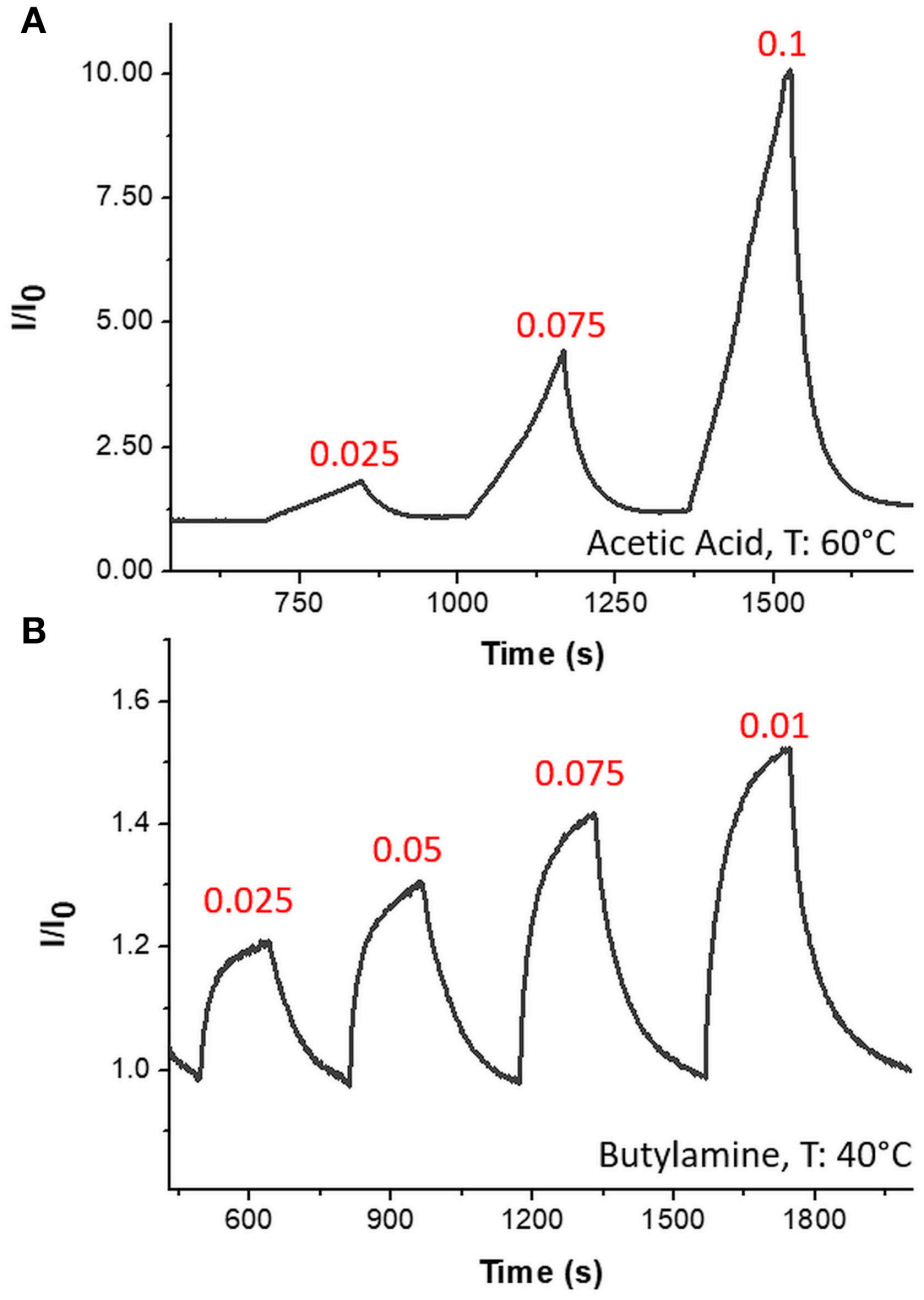
Transient response curves (Normalized current VS time) for Acetic Acid **(A)** and n-Butylamine **(B)**.

**Figure 9:**
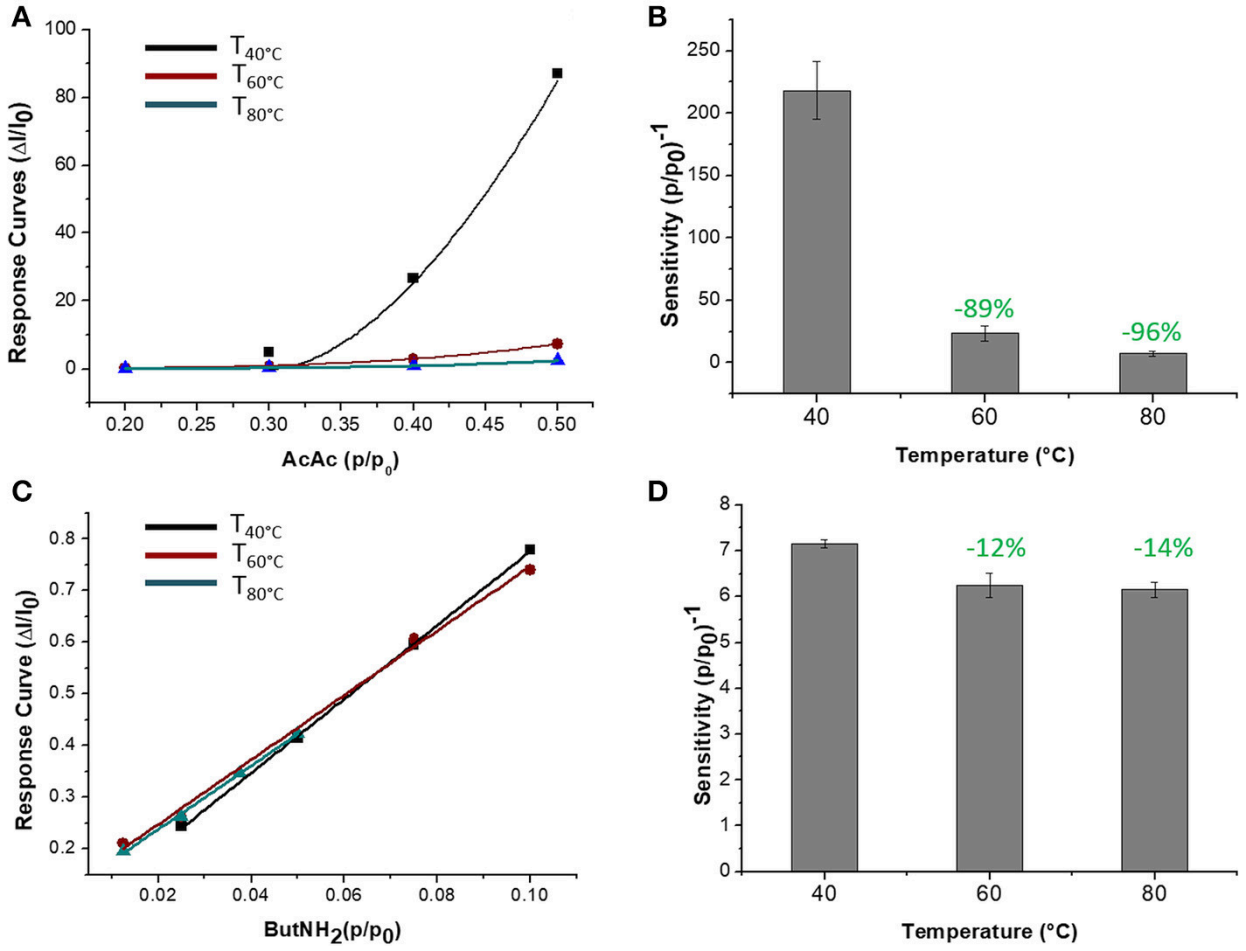
Response curves VS relative partial pressure at 40, 60, 80°C for Acetic Acid **(A)** and n-Butylamine **(C)** are depicted. Sensitivity of Acetic Acid **(B)** and n-Butylamine **(D)** were estimated.

**Figure 10:**
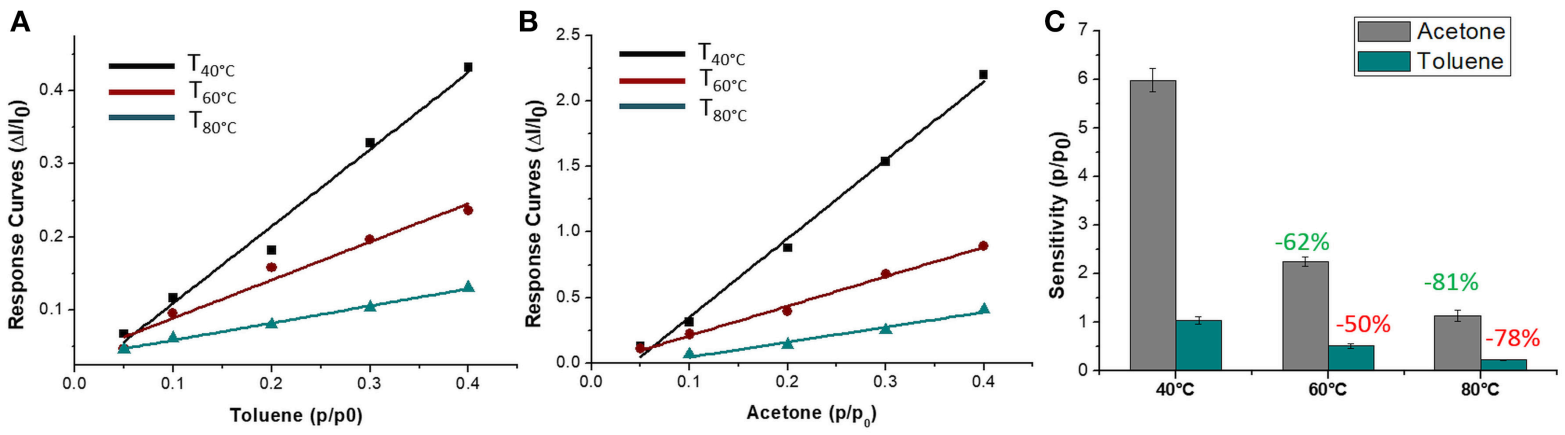
Response curves VS relative partial pressure at 40, 60, 80°C for Toluene **(A)** and Acetone **(B)** and corresponding sensitivity values **(C)**.

**Figure 11:**
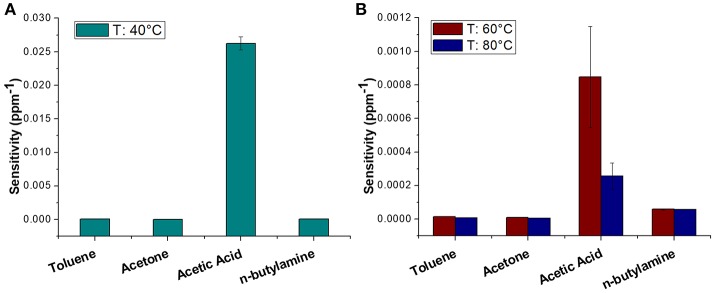
Diagram plotting sensitivity values (ppm^−1^) of Toluene, Acetone, Acetic Acid, n-Butylamine at 40°C **(A)** and 60, 80°C **(B)**.

The sensor was then exposed to known concentrations of VOCs belonging to different chemical classes. Thus, known amounts of vapors of n-butylamine, an aliphatic amine (weak base), and acetic acid carboxylic acid (weak acid) were flowed throughout the measuring chamber and the resulting current changes were depicted in Figure 8. The shape of the transient responses indicated that the sensor responded quickly to both the analytes (t^90^:≈130 s, where t^90^ is the time required by the sensor to reach 90% of the response) and it was regenerated in a few minutes (t^90^:≈150 s) by pure air. Further, the transient response shapes suggested two different VOC-surface rate of adsorption: very fast and with the reaching of a plateau in a few minutes for the amine (Langmuir-like kinetics) and linear without reaching an equilibrium phase for the acid. The kinetics of AcAc (Figure 8A) seems related to participation of multiple sites of interactions, possible lateral interactions between adsorbed molecules and a multilayer formation. The response curves too (Figures 9A,C), related to the current changes when the VOCs partial pressure increased, depicted different shapes within the measured ranges, meaning different affinity between the adsorbent fibers and the two VOCs. Specifically, they were linear for n-butylamine and linear, at low concentrations, to become exponential at higher concentrations for AcAc. The response curves to the increasing vapors of toluene and acetone, respectively, have been plotted in Figures 10A,B, showing curves with linear shapes, but different slopes (sensitivities). Transient response curves as the calibration curves are related to the ad/absorbing mechanisms that result in the chemical affinity of the VOCs to the material. The fibers are a heterogeneous system where MGC are the responsible of the electrical parameter: the adsorption of both polar and apolar VOCs onto mesopores (or structural defects) and planar surfaces of graphene, respectively, determine the changes in the charges density. The mesoporous structure could work as nucleation center for entrapping and growing molecules, like AcAc, with multiple functional groups. On the other hands, these organic compounds can provide conformational changes of the hosting polymer chains, thus contributing to the redistribution of the graphene network, which is responsible for the charge flow. Since all the VOCs induced a rise in current, the effect on network distribution inside fibers could be the dominant one. A comparison of the sensitivities to several VOCs and at different T_w_ is reported in Figure 11. When T_w_ increased to 60°C and then 80°C, the sensitivity to the amine (Figure 9D) decreased slightly (−12 and −14%, respectively), but enormously to the acid (Figure 9B) (−89 and −96%), acetone (−62 and −81%), and toluene (−50 and −78%) (Figure 10C). When compared to the other ones, the sensitivity to AcAc remained the highest at all temperature values, although the VOCs sensitivities ratios radically changed, reducing the global sensor selectivity (Figures 11A,B). Higher temperature seemed favor the permeation of amines despite the other VOCs, indeed the selectivity to n-butylamine lightly increased with the increase in the temperature, enhancing poor efficacy of a thermal role in the permeation and binding of the aliphatic amine with the fibers (Figure 11B).

**Figure 12:**
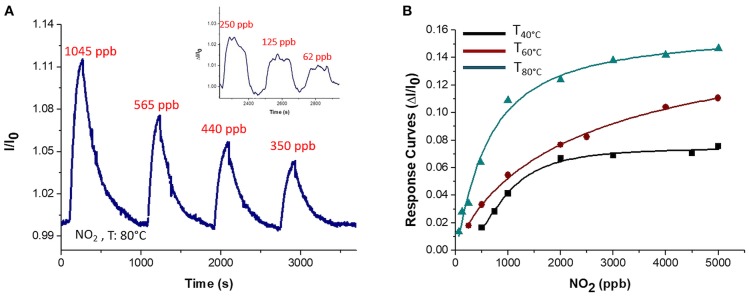
Transient response curve (Normalized current VS time) at 80°C **(A)** and Response curves VS concentration at 40, 60, 80°C for NO_2_ are depicted **(B)**. The inset in A showed an increase in current at lower concentration of NO_2_ (150, 125, 62 ppb).

Since graphene and overall graphene-oxide, has been investigated as highly sensitive materials to NO_2_ (Novikov et al., 2016) the fibrous chemosensor was exposed to increasing concentrations of this gas in air, ranging between tens of ppb and a few ppm. Response and recovery time occurred in a few minutes (Figure 12A) showing an abrupt increase in current as the gas entered the measuring chamber and a likewise decrease until the baseline when pure air was flowed (Figure 12A). More specifically, response and recovery time (t^90^) values were measured to be <40 and <100 s, respectively. Despite to the VOCs results, when T_w_ increased, the sensitivity to NO_2_ increased too, going from 3.91·10^−5^ to 5.7·10^−5^ and then to 1.16·10^−4^ ppb^−1^ at 40, 60, and 80°C, respectively (Figure 12B). Here the sensitivity was calculated as the slope of the curves in the linear range (i.e., at lower concentrations). The affinity of the chemosensor to NO_2_ is depicted by the Langmuir-like calibration curves, as well as the significant effect of the temperature to the gas detection (Figure 12B). Since NO_2_, that is an electron withdrawing, causes an increase in current, the MGC inside fibers is supposed to perform as a p-type semiconductor, delivering electrons to the gas molecules, leading an increase of hole concentration and leading so to an increase of graphene's conductivity. The high polymer porosity seems to allow the gas diffusion. Further the sensitivity at 80°C was valued to be 4 times higher than at 40°C. The increase in sensitivity could be due to the redistribution and orientation of graphene within polymer fibers due to the heating, allowing the gas adsorption onto a larger number of exposed binding sites, despite of the unfavorable energies involved in the phenomena of ad-adsorption. The LOD_80°C_ (defined as 3 ^*^ standard deviation of the blank) has been calculated to be ~2 ppb. In literature, chemoresistors based on graphene hybrids, reported limits of detections ranging between 10 ppm and 64 ppb at room temperature (Latif and Dickert, 2015).

## Conclusions

The present study reported the development of a conductive nanofibrous and nanocomposite polymer sensor designed so that its sensitivity and selectivity could be regulated by temperature. Electrospinning technology allowed, in one step and in 2 min, the fabrication of rough fibers comprising two thermoplastic polymers and nanopowder of mesoporous graphitized carbon. Indeed, exploiting the peculiarity of the deposition technique, through the usage of two incompatible polymers (PS and PHB), a proper mix of organic solvents and a surfactant salt, the resulting fibrous layer comprised a wide adsorption surface and a lot of interfaces that are likely features for chemical sensors. The selected amount of MGC (0.93% mass ratio), subjected to electrospun deposition together with the two polymers, appeared both finely spread inside fibers and more densely packed in some dots. Such a MGC concentration was the minimum concentration allowing the generation of stable and reproducible electrical signals at room temperature. Since the electrical conduction of nanocomposites is related also to their state of dispersion and their orientation inside the polymer, the heating of fibers, by improving the connectivity of the conductive networks, caused a non-linear increase in current of the sensor. The sensor was able to work in a stable and reproducible way between 40 and 80°C without any significant degradation. Among the various chemical classes of the tested VOCs (aliphatic amine, aromatic hydrocarbons, ketone, and organic acid) the sensor resulted highly sensitive and selective to acetic acid. When fibers were heated, the sensitivity to the acid fell down, decreasing by 96% at 80°C if compared to that of the sensor at 40°C. On the other hand, although an increase in temperature caused a general decrease in sensitivity, higher temperature affected only slightly the amine permeation, thus modifying the partial selectivity of the sensor. Water vapors, too, were ad-absorbed by the fibrous layer, probably due to the porous and extremely wrinkled structure of the film, the number of interfaces of the heterogeneous fibers, and the mesoporous structure of MGC. An increase in temperature (from 40 to 80°C) caused a reduction in sensitivity by more than half. A completely different effect was reported when the sensor was exposed to traces of NO_2_ and then heated. When temperature increased, the sensitivity to NO_2_ increased too, until it achieved a LOD of about 2 ppb, when the sensor worked at 80°C. The high polymer porosity favored the gas diffusion such as the high available surface area of MGC increased the chance to bind the analyte. The further increase in sensitivity due to the heating could be presumably caused by the redistribution and orientation of graphene within polymer fibers that in turn could occupy a larger number of binding sites. The general decrease in sensitivity to all the VOCs and the relative humidity at higher temperature values despite the increasing sensitivity to NO_2_, means that the sensor can be tuned in order to be more selective to the gas and that the role of the potential interferents in complex environments can be significantly lowered. Further studies are needed to understand the whole mechanism of ad-absorption occurring between MGC and the VOCs/gas as well as the role of each polymer inside fibers when the working temperature changed. However, this preliminary study suggests that temperature can be a useful parameter for modulating the selectivity of defined nanocomposite polymeric sensors, letting us suppose that, as it happens for the metal oxide sensors, the same sensor could be designed to work successfully in an array, simply changing its working temperature.

## Data availability statement

The raw data supporting the conclusions of this manuscript will be made available by the authors, without undue reservation, to any qualified researcher.

## Author contributions

AM contributed to the conception and design of the study. EZ contributed to the definition of electrospun parameters. JA carried out electrospinning deposition and laboratory measurements. EZ provided the electronics of the measuring system. AB designed the water vapor measurements set-up. FD, GS-M, JA, AM, and EZ contributed to the graphics and data treatment. GV provided the EPR analysis. All authors contributed to manuscript revision, read and approved the submitted version.

### Conflict of interest statement

The authors declare that the research was conducted in the absence of any commercial or financial relationships that could be construed as a potential conflict of interest. The handling editor and reviewer DK declared their involvement as co-editors in the Research Topic, and confirm the absence of any other collaboration.

## Footnotes

^1^https://www.electrochem.org/dl/interface/wtr/wtr10/wtr10_p035-040.pdf

^2^https://sites.google.com/site/miller00828/in/solvent-polarity-table.

## Abbreviations

EPR: Electron Paramagnetic Resonance
ES: Electrospinning
IDE: Interdigitated Electrode
LOD: Limit of Detection
MGC: Mesoporous Graphene Carbon
PHB: Polyhydroxibutyrate
PS: Polystyrene
sccm: standard cubic centimeter per minutes
SEM: Scanning Electron Microscopy
TEM: Transmission Electron Microscopy
VOC: Volatile Organic Compound.
